# High-Throughput Behavioral Screens: the First Step towards Finding Genes Involved in Vertebrate Brain Function Using Zebrafish

**DOI:** 10.3390/molecules15042609

**Published:** 2010-04-12

**Authors:** Robert Gerlai

**Affiliations:** Department of Psychology, University of Toronto Mississauga, 3359 Mississauga Road North, Rm 3035, Mississauga, Ontario L5L 1C6, Canada; E-Mail: robert_gerlai@yahoo.com; Tel.: +1-905-569-4255; Fax: +1-905-569-4326.

**Keywords:** zebrafish, high-throughput behavioral screening, fetal alcohol syndrome, alcoholism, learning and memory, fear and anxiety

## Abstract

The zebrafish has been in the forefront of developmental biology for three decades and has become a favorite of geneticists. Due to the accumulated genetic knowledge and tools developed for the zebrafish it is gaining popularity in other disciplines, including neuroscience. The zebrafish offers a compromise between system complexity (it is a vertebrate similar in many ways to our own species) and practical simplicity (it is small, easy to keep, and prolific). Such features make zebrafish an excellent choice for high throughput mutation and drug screening. For the identification of mutation or drug induced alteration of brain function arguably the best methods are behavioral test paradigms. This review does not present experimental examples for the identification of particular genes or drugs. Instead it describes how behavioral screening methods may enable one to find functional alterations in the vertebrate brain. Furthermore, the review is not comprehensive. The behavioral test examples presented are biased according to the personal interests of the author. They will cover research areas including learning and memory, fear and anxiety, and social behavior. Nevertheless, the general principles will apply to other functional domains and should represent a snapshot of the rapidly evolving behavioral screening field with zebrafish.

## 1. Introduction: Why Should We Use Zebrafish?

There are several laboratory organisms with which one can investigate such complex questions as how genes influence behavior or how certain biochemical processes affect the functioning of the brain. Pharmaceutical research companies as well as academic laboratories have been using rats in their preclinical investigation to screen libraries of compounds to identify drugs that may be beneficial in a range of human brain and behavioral disorders. The house mouse has been utilized also in countless studies in which the effects of specific genes (reverse genetics) have been investigated as to how they alter behavior and other levels of brain function [1,2]. Simpler laboratory organisms such as the flat worm [3], the sea slug [4], or the fruit fly [5] have also been successfully employed to model and study certain genetic aspects of brain function and behavior. Why then is there increasing interest in zebrafish?

The zebrafish appears to strike a good compromise between system complexity and practical simplicity. It is a vertebrate species with a physiology [6], brain anatomy [7] and neurochemistry [8] fundamentally not different from characteristics of our own species. Most importantly, significant nucleotide sequence homology (70–80%) has been shown between zebrafish and human genes, and the amino acid sequence homology of functionally relevant domains of proteins has been found to be even more evolutionarily conserved [9,10]. Briefly, there is reasonable hope that if one identifies a gene involved in particular functions/dysfunctions of the zebrafish brain, there will be a human homolog that may also serve similar functions. Thus the translational relevance of zebrafish research may be high.

## 2. Genetic Tools for Zebrafish Abound

Excellent reviews have been published on the numerous genetic approaches that are available for zebrafish [11,12,13,14] and I will not reiterate here these techniques in detail. Suffice it to say that both reverse genetic and forward genetic methods are available. The former aims at studying the phenotypical effects of the targeted manipulation of known genes. The latter wants to discover novel genes by the introduction of random mutations. For instance, among the reverse genetic tools, TILLING has been employed successfully [15] and antisense oligonucleotide knockdown methods using morpholinos [16] has also been employed. Forward genetic studies have been able to utilize the classical chemical mutagen, ethyl nitrosourea (ENU), and the high resolution markers developed for zebrafish now allow fine mapping and linkage analysis-based positional cloning of the mutated genes [17]. In addition to ENU-based mutagenesis, other methods have also been employed. Viral-vector mediated insertional mutagenesis, although not without disadvantages, has had the major advantage over ENU as it bypasses the labor intensive linkage analysis-based positional cloning: the viral tag left behind by the vector can be easily identified and thus the gene can be cloned using faster molecular methods [11]. Other genetic methods, e.g. zinc-finger-based knock-out of genes [18] or a gene-breaking transposon-based method to generate mutations [19] have also been developed for zebrafish.

Forward genetic approaches have been particularly successful with zebrafish especially as compared to similar methods with the classical biomedical research organism, the house mouse, for one simple practical reason: the number of subjects one can generate and study is orders of magnitude higher in case of the zebrafish as compared to the mouse. 

## 3. The Number Game: Characteristics of Zebrafish Ideal for High Throughput Screening

The number of animals one can screen is a crucial factor in forward genetics where one does not know which and how many genes may influence the phenotypical function in question. In case of brain disorders or behavioral function, the number of such genes may be quite large and thus one has to screen thousands of mutants to tackle this complexity and identify appropriate mutations, which than may lead to the identification of the genes involved. One can of course generate the same number of zebrafish and mice for screening purposes. However, there are several reasons why zebrafish may be preferred. First, a single female can produce 200 offspring at every spawning and can spawn multiple times a week. Second, zebrafish is small (4 cm long) and is highly social and thus a large number of subjects may be housed cheaply in a small animal holding room. For example, a standard zebrafish stand-alone high density rack system (e.g. Aquatic Ecosystems Inc, FL, or Aquaneering Inc. CA) with six shelves and about twelve 3 liter tanks per shelf, can house 2,000–3,000 zebrafish, and a 40 m^2^ standard vivarium room may be fitted up with 5-6 such racks. That is, the same room that may house a couple of hundred mice can have close to twenty thousand zebrafish in it. 

## 4. Behavioral Test Paradigms: The Bottle Neck of Zebrafish Research 

Although zebrafish has numerous advantages over other laboratory organisms, in one important regard they appear significantly inferior. Very little is known about the behavior of this species and there are only a few behavioral test paradigms available [20]. Why is this a problem? One can analyze brain function directly and behavior may be inferred from endophenotypes, neurobiological phenomena below the level of behavior, some may argue. This may be true, although appropriate neurobiological correlates, surrogate measures of behavior, and whether they exist, are hotly debated topics. Furthermore, when the question concerns identification of novel genes or novel pharmaceutical compounds, I and others have argued that behavioral analysis is perhaps one of the better approaches (for a review see [21]; also see [22]). This is because behavioral analysis can potentially probe a broad spectrum of brain functions in a large number of subjects efficiently. It is not limited to particular brain regions or particular neurobiological mechanisms and it is relatively simple and cheap to conduct. Briefly, high throughput behavioral screens have the potential to reveal numerous mutation or drug induced functional changes in the brain. Although the behavior of zebrafish has been studied for decades, the number of behavioral studies has been orders of magnitude below those conducted with mice, rats, or other classical laboratory species including the fruit fly [20]. Nevertheless, for the past few years a clear upsurge of zebrafish behavioral studies is evident, indicating that behavioral neuroscience and behavior genetics has started acknowledging the advantages of this species. Here I review some of these recent studies focusing on the question of how behavioral analysis may be made high throughput and how high throughput behavioral paradigms may facilitate the discovery of novel genes and compounds affecting brain function. Three main behavioral focus areas will be represented in this review: learning and memory, fear and anxiety, and social behavior. These areas represent a personal bias, of course, but I hope they will sufficiently illuminate the utility of zebrafish in neuro-behavioral genetic research. 

## 5. Learning and Memory

Learning and memory has been extensively studied by scholars of several scientific fields. Numerous mechanistic questions related to how learning occurs and what memory is have been successfully tackled. For example, by now a large number of genes and biochemical mechanisms underlying learning and memory have been identified [23]. Is there any utility for zebrafish in this research, a newcomer in this well advanced field? Although hundreds of genes involved in learning and memory have indeed been identified, we are far from being able to understand the mechanisms of these complex processes. There may be a large number of undiscovered genes whose protein products represent unknown but crucial players in learning and memory. For example, according to conservative estimates, most vertebrate genomes contain about 30,000 genes. Recent microarray studies suggest that at least 50% of all the genes of the genome are expressed in the vertebrate brain (e.g. in zebrafish), *i.e.,* about 15,000 genes [24]. Given that plasticity is perhaps the most complex aspect of brain function, it is likely that a large proportion of these genes, *i.e.,* thousands of them, are involved in one way or another in neural mechanisms subserving plasticity, *i.e.,* learning and memory. How can one identify such a staggering number of genes? Mutagenesis studies have been successfully performed with mice and have led to the discovery of novel genes, but as explained above, such forward genetic studies are significantly limited by the relatively small number of mice one can study. Zebrafish enjoys a major advantage here. 

A number of laboratories have realized this and thus the development of high throughput learning paradigms specifically designed for zebrafish is well on its way. Investigators have started to characterize the cognitive capabilities of zebrafish and have already published on the first test methods that can measure learning and memory efficiently and fast (for examples see [20]). The key in these paradigms concerns automatability. Even if one needs to employ several repeated training trials, if these trials can be administered in an automated manner, and if the behavioral responses that reflect learning and memory performance are easy to measure and do not require the constant presence and attention of an experimenter, the paradigm may be run in multiple test apparati in parallel and thus become high throughput.

**Figure 1:**
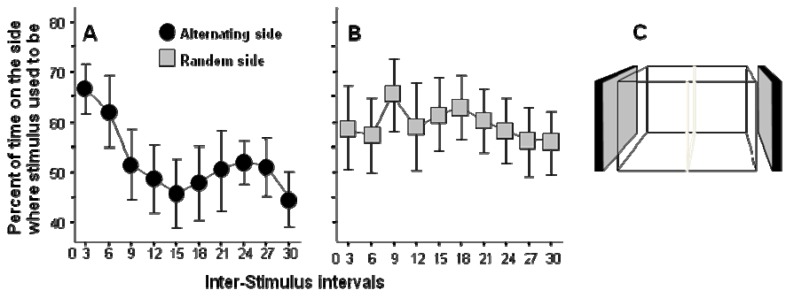
Performance during the Inter-Stimulus Intervals (ISI): percent of time test fish spent on the side of the experimental tank where the stimulus fish used to be shown (modified from [25]).

A successful high throughput learning task design has been published [25]. In this task zebrafish were exposed to the sight of moving (animated) images of conspecifics shown on a computer screen placed by each side of the experimental tank. Previously, access to view a shoal (group) of zebrafish has been shown to have rewarding properties for experimental zebrafish and that this visual stimulus (the sight of a group of zebrafish) can support good learning performance [26]. Subsequently, it has been demonstrated that even computer animated moving images of zebrafish (each life size “member” of this artificial shoal moves realistically in different directions) can serve as a strong positive stimulus [27]. This simple technique allowed the development of an automated high throughput task [25]. The task is also simple. For a short period of time (20 seconds in reference [25]) the image of the moving shoal is shown and then it is turned off for 90 seconds. After this 90-second no image period the image of the moving shoal is shown again on the opposite side of the test tank for 20 seconds, and the sequence repeats itself. As a result of the alternating image presentation sides, zebrafish have to make a choice during the no-image period as to whether they stay close to the side where the image was just shown, or move to the opposite side, where the image will appear. The natural tendency of zebrafish is to stay close to their conspecifics and thus initially spend the highest amount of time near the side where the image was shown last. However, as the training proceeds, zebrafish spend increasingly longer amount of time near the side that will show the image (Figure 1). There are several important points to make about these results. First and foremost, the motivation to stay close to conspecifics does not change (habituate) over time and thus the experimental subjects continue to perform in this task. This is a major advantage over other motivators, including food, which has been difficult to work with especially in the case of zebrafish, which gets food satiated quickly and can stay healthy without food for several days. Second, the stimulus is a visual cue which can be administered precisely and using consumer grade (reasonably priced) video-equipment. Third, the behavioral response (distance from stimulus screen) is easy to quantify using video-tracking systems and/or motion detectors (e.g. photocell detector arrays). Fourth, multiple trials (in reference [25] thirty trials) can be administered without the intervention by the experimenter. The fish stays in the test tank and is given the stimuli and their responses are measured repeatedly across the continuous sequence of trials. As a result of all these above features, the paradigm is fully automated and thus multiple set-ups can be run in parallel. Although the 30 trials required 3,300 seconds (55 min) per experimental fish [25], one can easily set up several such test apparati. Briefly, the throughput of the task depends only upon how many computer screens and test tanks one can fit into a test room. In our facility a 20 m^2^ test room could be easily fitted with 50 such test tanks. That is, in an eight hour work day, one can test 400 zebrafish in a single room using this learning task, a sufficiently high throughput even for large scale mutagenesis screens [28]. 

There are, of course, numerous unexplored questions regarding the above test paradigm. For example, do zebrafish forecast the future: is their performance improvement due to better timing of their responses (knowing when and where the shoaling image will appear in the near future)? Or is their performance improvement simply due to acquisition of CS-US association: disappearance of the stimulus on one side (the conditioned stimulus) predicts the reappearance of the unconditioned stimulus (the shoal image) on the opposite side. There are many questions about the optimization of the task as well: is the 20 seconds/90seconds stimulus/no-stimulus interval length the best? Could longer tanks (the original experiment was conducted in a 50 cm long tank, a distance that can be easily traversed by the fast zebrafish) be more appropriate allowing more sensitive detection of performance improvements/deficits? Also, how would other stimulus/no-stimulus schedules (random *vs.* fixed ratio, increasing stringency *vs.* constant) affect the behavior of the fish? Last, mechanistic questions as to what neuroanatmical structures subserve the task, what drugs may influence performance in it, and how sensitive it may be to detect mutation induced changes all will have to be explored. Clearly, there are many questions when one introduces a new paradigm. Nevertheless, the above example demonstrates how one can utilize species-specific perceptual, motor and motivational characteristics to design relatively simple and high throughput behavioral test methods that may allow addressing many of the above questions in the future. 

The above learning task have an important temporal component, the delay between the stimulus presented on one vs. the other side of the tank and as such may allow the analysis of a complex forms of learning known to be associated with the mammalian hippocampus, trace conditioning [29] and/or acquisition of relational memory [30]. Although fish do not have a structure whose circuitry resembles that of the mammalian hippocampus, they do possess a brain region, the lateral pallium that is believed to be a structure homologous to the mammalian hippocampus [31]. Furthermore, fish without that classical mammalian hippocampal circuitry have also been found to be able to learn spatial learning paradigms, a class of tasks that is associated with hippocampal function in mammals [32]. Spatial learning has also been demonstrated in zebrafish [33], however, the spatial task employed (learning to find a particular location in a plus maze) was extremely time consuming. It required many repeated trials which could be administered only manually. Could one design a high throughput spatial task for zebrafish? 

This question was answered in a recent study [34] that demonstrated good learning performance of zebrafish in a latent-learning paradigm. The paradigm consisted of two phases, a long training phase and a brief probe trial. During the training phase zebrafish were allowed to explore a complex maze which consisted of a starting chamber that was connected to a goal chamber by a left and right tunnel. Zebrafish were allowed to explore the maze in groups of ten (a shoal) for 16 consecutive days, each day once for 50 min. Allowing zebrafish to swim around the maze in ten-member shoals facilitated active exploration and reduced passive fear responses. During the exploration of the maze certain shoals were allowed to go through only one of the tunnels, *i.e.,* there was a set of fish for which only the left tunnel was open and the right tunnel was blocked and another for which the left tunnel was blocked and only the right tunnel was open, and yet another group for which both tunnels were open, a spatial exploration task. The second part of the paradigm was a short (10 min long) probe trial, during which both tunnels of the maze were open, a shoal of stimulus fish was placed inside a transparent container and into the goal chamber of the maze, and the experimental fish were tested singly in the maze. Given the highly social nature of zebrafish, the experimental subject was highly motivated to get as close to the stimulus fish in the goal chamber as possible. Which route, the right *vs.* the left tunnel, the experimental fish took was video-recorded and analyzed. The results showed that those fish that experienced the right tunnel open during the maze exploration phase of the paradigm also used the right tunnel during the probe trial, those fish that experienced the left tunnel open used the left tunnel during the probe trial and those fish that experienced both the left and right tunnel open chose randomly. Why is this paradigm high throughput? Although the exploration phase of the paradigm took 16 days, 10 fish could be exposed to the maze each time. Because these fish are not monitored and their behaviour do not need to be analyzed, one could set up a large number of mazes and train a large number of fish every day. The probe trial is conducted for every fish separately, but it lasts only for 10 min and the swim path of the fish can be quantified using automated video-tracking techniques [35]. Thus this phase of the paradigm can also be made high throughput. Furthermore, given the spatial nature of the task, this paradigm is likely to be capable of tapping into complex forms of learning and memory. 

There are again many questions about this novel paradigm. What motivates the fish to learn the maze? In other words, why fish remember the tunnel they explored before? This form of learning is termed latent learning because apparently there is no external experimenter controlled motivator (positive or negative reinforcement) presented. However, it has been argued [34], based on prior supporting evidence, that exploration of novelty itself is rewarding in this task, and the novel aspect of the maze is what keeps the fish motivated to explore and learn. The results of this study also suggested that learning in this paradigm is likely based upon acquiring and remembering external visual cues, *i.e.,* spatial learning, a hypothesis that will need to be proven in the future. But again, despite the novel aspect of the task and the fact that there may be numerous questions one could explore with it, the paradigm does appear to be appropriate for high throughput screening of learning and mnemonic characteristics of zebrafish and mutation induced changes in these characteristics. 

There are numerous human disorders associated with memory loss and/or impairment of cognitive function, perhaps the most devastating and prevalent is Alzheimer’s disease. But milder forms of memory problems, MCI (mild cognitive impairment) and age-related memory loss, also affect a large percentage of the aging human population in the 21st Century. Given the large unmet medical need associated with these diseases and the potential complexity of the genetic mechanisms underlying them [36], the importance of appropriate screening tools with which mutation induced changes in learning and memory processes may be identified is unquestionable.

## 6. Fear and Anxiety

Learning and memory are not the only complex phenomena and their dysfunction is not the only large unmet medical need. Fear and anxiety also affects a large percentage of the human population [37] and despite concerted efforts by pharmaceutical research companies and academic laboratories and despite the existence of several drugs, proper treatment is still not available for a large proportion of patients. This is mainly because we still do not understand the biological mechanisms of fear and its exaggerated or misdirected forms, anxiety. Behavioral paradigms that could induce fear and allow its proper quantification should facilitate discovery of these mechanisms using model organisms. It has been argued by several researchers that zebrafish may be an excellent tool for this purpose [38]. Fear responses are perhaps some of the best studied behavioral characteristics of this species. Instead of reviewing this rich literature thoroughly, I will focus on the latest developments that are most relevant for our current discussion: high throughput mutagenesis screening. 

**Figure 2:**
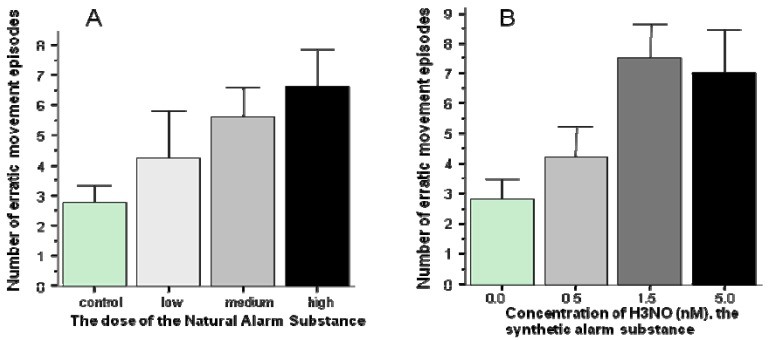
The natural alarm substance (Panel A) as well as the synthetic alarm substance, H3NO (Panel B) elicits increased number of erratic movement responses (modified from [39,40]).

Fear responses have been most reliably induced in zebrafish using a chemical cue, the alarm substance [39]. Alarm substances have been shown to elicit fear and panic reactions in a broad range of fish species [39]. These substances, which occur naturally in the skin of many fish species, are released when the skin is cut or damaged. In nature, the alarm substance is believed to signal danger, perhaps the presence of an actively hunting predator (piscivore fish species or a bird of prey). In the laboratory, the alarm substance has been successfully utilized to experimentally induce fear responses. Zebrafish have also been shown to reliably respond to this chemical cue with alarm reactions that include erratic movements (zig-zagging), jumping (or leaping) and freezing (complete immobility) [39]. From the perspective of mutagenesis screening, however reliable these responses may seem, the alarm substance approach suffered from a major disadvantage. This substance had to be extracted from the skin of conspecifics which entails cutting or homogenizing the skin of freshly sacrificed fish and washing, diluting the extract. Because of the variability inherent in this extraction process (how deep the cut was, whether blood was also drawn, whether the epidermal club cells producing the substance were sufficiently damaged, interindividual differences in the donor fish, *etc.*), the exact dose and potency of the substance could not be ascertained across multiple experiments (multiple extractions). Most recently, however, zebrafish has been shown to respond to a synthetic alarm substance that shares a key chemical structural element with that of natural alarm substances from several fish species [40]. Hypoxantin-3-N-Oxide, H3NO, was found to induce alarm reactions in zebrafish similar to those elicited by the natural alarm substance [40] (Figure 2). Thus, it is now possible to precisely control the dose of the alarm substance and reduce unwanted experimental error variation, a crucial requirement for high throughput mutagenesis screens.

Although the synthetic alarm substance hypoxanthine 3-*N*-oxyde (H_3_NO), now allows precise and replicable fear induction, this method suffers from a drawback. Olfactory cues are notoriously difficult to work with. Although the onset (delivery) of the cue and now also the dose can be precisely controlled, its offset (washout) is difficult to achieve. In most behavioral paradigms, experimenters want to introduce the subject to its test chamber (tank) and let the subject habituate, establish a stable baseline behavior before administering the cue (the alarm substance in this case). This allows pre- and post-cue delivery periods to be compared and thus is a more powerful experimental design. Ideally, after the delivery of the cue and recording the effects of this delivery, one would like to turn the cue off and again compare periods during and after cue delivery. But this is not possible, or quite cumbersome, with olfactory cues. Furthermore, even if the experimental paradigm does not require turning off the cue during the behavioral session, the cue may be difficult to remove from the tank for the next subject. Residual amounts of the alarm substance may remain in the test tank even after emptying and refilling the test tank. As even trace amounts of the alarm substance may influence the behavior of the fish, this olfactory cue is difficult to work with especially when one wants to run a large number of fish as required for mutagenesis screening.

To circumvent the above issues, cues of other modalities may have to be tried for the induction of fear responses. One obvious modality is vision. Zebrafish, being diurnal vertebrates, have excellent vision and may respond well to visual cues. Indeed, zebrafish have been demonstrated to respond differentially to the sight of live fish according to whether the fish species shown were predatory or harmless and whether they were sympatric (coinhabiting the geographical region) or allopatric with zebrafish [41]. The latter study also demonstrated that zebrafish uniquely responded to a sympatric predator, the Indian leaf fish (*Nandus nandus*) and the sight (solely visual stimuli) of the predator is sufficient to induce a maximal fear response (erratic movements and jumps). Utilizing this finding, subsequently zebrafish have been found to exhibit significant antipredatory responses not only to the sight of live Indian leaf fish but also to animated (moving) computer images of this species [42]. In this latter paradigm, both the presentation of stimuli and the recording and analysis of the fear responses were conducted in an automated computerized manner, *i.e.,* the test paradigm was scaleable and thus high throughput. Although numerous parameters of this first automated fear paradigm will have to be optimized (e.g. size of the test tank, size and speed of movement of the predator image, presence or absence of hiding places, level of illumination, *etc.*), the results demonstrate the feasibility of high throughput screening for agents (mutations or pharmaceutical compounds) that may have fear altering properties.

## 7. Social Behavior

The last behavioral focus area I review in this paper is social behavior. Social behavior is a common term for a large number of complex behavioral phenomena ranging from agonistic (aggressive) encounters to reproductive (courtship) behaviors. The current review focuses on a behavior within this broad range termed affiliative behavior, social cohesion or group forming behavior. Affiliative or group forming behaviors are characteristic of our own species. Humans tend to form groups, which in modern history led to the development of the complex society where a set of rules govern our behavior. We are particularly sensitive to social signals and tend to spontaneously follow a large number of complex social rules. Briefly, being social and being sensitive to social signals is an inherent human trait. There are numerous human disorders that are associated with abnormalities in social behavior, a well known example of which is the autism spectrum disorders or ASD. Treatment for ASD and other forms of abnormal social behaviors is lacking for two main reasons. One, the mechanisms underlying these diseases are unclear. Two, the mechanisms underlying social behaviors in general are not understood. Laboratory model organisms have been proposed to speed up the discovery of such mechanisms. The question as to whether autism may be modeled using animals is complicated. Some may be skeptical and say that in order to model autism in animals one would need to understand its mechanism first in humans, so what’s the use of animal research? Nevertheless, it is becoming increasingly clear that even such complex phenomena as social behavior has not only face but also construct validity, *i.e.,* not only looks similar in animals but may also be mechanistically similar to that of our own species. Briefly, it may make sense to study social behavior in vertebrates other than humans, discover the underlying mechanisms in the laboratory organism, and look for translational aspects of the work, *i.e.,* human homologues. Zebrafish is perhaps the most social vertebrate model organism currently under study in the laboratory. Zebrafish are found swimming in groups in nature, a behavior that they maintain under the artificial confines of the laboratory [43]. It is this swimming together response, or shoaling, that may be an excellent behavioral phenomenon to study from a translational perspective. Answering such questions as to what neurobiological mechanisms (circuits, synaptic processes, biochemical interactions) underlie group forming or social cohesion in zebrafish may help us understand human social behavior and ultimately perhaps the mechanisms of the abnormalities of human social behavior. The first step in this research could be the characterization of social behavior in zebrafish followed by the development of behavioral test paradigms that could detect mutation or drug induced changes in brain function at the level of social behavior. Below I present some examples of recent discoveries with zebrafish that may be useful to make the first steps in this direction. 

Zebrafish forms groups and swims in group formation but due to unavailability of appropriate behavioral quantification methods, the complexities of this behavior have not been properly described. Recently, a method has been designed that allows the quantification of numerous parameters of the shoaling behavior, including moment to moment changes of the distances among every possible pairs of fish within the shoal [44,45]. This method led to the discovery of a periodic (cyclical) fluctuation of shoal cohesion [45]. The method is now further developed to allow high throughput automated tracking of multiple fish and thus the precise description of how the entire shoal behaves. The method may enable one to screen for mutations but would require to generate a group of fish that carry the same mutation, which would necessitate breeding an extra generation (e.g. testing not the individual mutant fish but its offspring). Perhaps a faster behavioral screening method would be if single fish and its response to social stimuli could be tested. The disadvantage of the latter approach is that complex group dynamics may not be detected but the advantage is that the test would save the extra generation of breeding.

**Figure 3:**
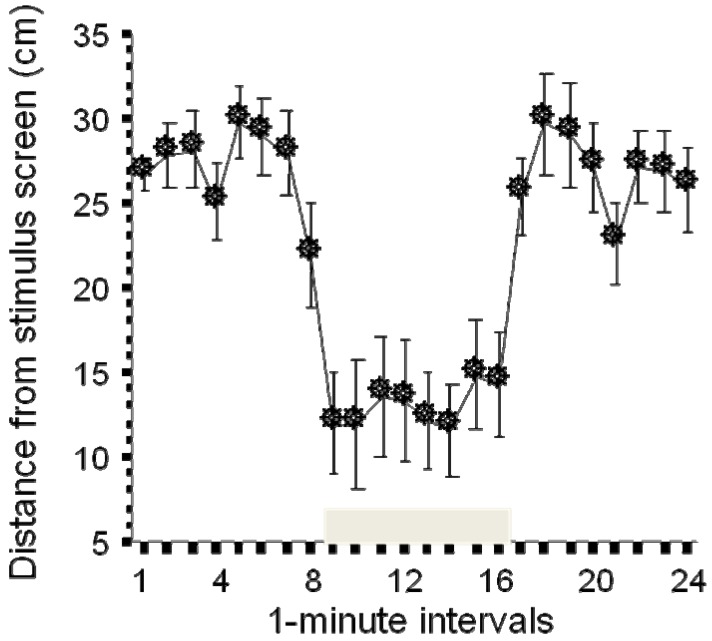
Presentation of animated (moving) images of zebrafish (grey horizontal bar above the X-axis) induces a significant shoaling response: upon the presentation of the images, the experimental zebrafish moves closer to the presentation screen (modified from [27]).

*Note*: When the stimulus is turned off, the distance of the experimental zebrafish from the stimulus screen returns to baseline. Mean ± SEM are shown.

Testing individual responses to social stimuli has been achieved with an experimental set up similar to the predator visual stimulus paradigm [27]. Here the computer monitor placed on the side of the experimental tank shows animated (moving) images of zebrafish (five fish in this case). Each fish on the monitor moves independently and in different randomized directions and with a speed that changes from second to second while remaining within the range of a normally swimming zebrafish. This artificial “shoal” elicits a robust behavioral response (Figure 3). The single experimental fish placed in the test tank usually does not exhibit a preference for any sides of the tank, explores the entire tank, and thus its position when averaged over a period of time (e.g. for one minute intervals) ends up to be in the middle of the tank, which is 25 cm away from the computer screen in case of a 50 cm long experimental tank. However, as soon as the computer screen shows the artificial shoal, the experimental zebrafish moves closer to the computer screen and on average stays about 10 cm away from it, a distance that is in line with what has been obtained with freely moving zebrafish in a real shoal [44,45]. Given that the visual stimulus that elicits the response is computer controlled and the subject’s distance from the stimulus screen is recorded using computerized video-tracking, the entire test paradigm is automated, *i.e.,* does not require the presence of the experimenter during the behavioral recording session. The paradigm therefore is high throughput and has utility in screening for mutation or drug induced changes in social behavior. Indeed, this paradigm has been already used to detect strain (genetic) differences between populations of zebrafish, and alcohol and dopamine receptor antagonist induced changes in social behavior ([27] and unpublished results).

## 8. Concluding Remarks

The above discussed behavioral paradigms represent just a snapshot of the possibilities regarding how behavioral analysis could be utilized for the identification of mutation or chemical compound induced changes in brain function in zebrafish. Concerted efforts are being made in several zebrafish behavioral neuroscience laboratories to develop such novel test paradigms. Some of these paradigms have already been published, suggesting that the future of phenomics (phenotypical screening applications, [21]) is quickly becoming a reality. Whether these phenotypical screening tools will lead to the identification of translationally relevant genes (molecular targets) and drugs that will advance our understanding of human brain diseases and dysfunctions, is a question that will only be answered once the screening tools have been put to actual use. However, given the fact that behavior is the output of the brain and that vertebrates share numerous biological features, including nucleotide sequence homologies, it is likely that zebrafish neurobehavioral genetics will facilitate the identification of numerous genes and compounds leading to the understanding and better treatment of human brain disorders.

## Figures and Tables

Graphs show the performance of fish averaged over three successive ISI’s for a total of 30 intervals. Mean ± SEM are shown. Note that during the ISI (90 seconds each) no stimulus fish are shown on either side. Note the results suggest that experimental zebrafish under the alternating condition (Panel A) learned to reduce the time spent on the side of the tank where the stimulus was shown before and increased the time spent on the opposite side where the stimulus was going to appear but when the stimulus was presented randomly (Panel B) no such learning occured. Panel C shows the basic experimental set up with the two stimulus screens on the side of the tank and the small frame divider in the middle of the tank indicated.

Mean ± SEM are shown. Note that the precise concentration of the natural alarm substance, which was extracted from the skin of zebrafish, could not be determined and thus only the relative (compared to the stock solution) doses can be indicated. Also note the comparable level of erratic movement responses to the natural and the synthetic alarm substances.
